# Stroke Affected Lower Limbs Rehabilitation Combining Virtual Reality With Tactile Feedback

**DOI:** 10.3389/frobt.2020.00081

**Published:** 2020-07-09

**Authors:** Alexander V. Zakharov, Vladimir A. Bulanov, Elena V. Khivintseva, Alexander V. Kolsanov, Yulia V. Bushkova, Galina E. Ivanova

**Affiliations:** ^1^Department of Neurology and Neurosurgery, Samara State Medical University, Samara, Russia; ^2^MathBio Laboratory, IT Universe Ltd., Samara, Russia; ^3^Institute for Innovative Development, Samara State Medical University, Samara, Russia; ^4^Research Center of Cerebrovascular Pathology and Stroke, Ministry of Health of the Russian Federation, Moscow, Russia

**Keywords:** stroke, rehabilitation, virtual reality, tactile feedback, biofeedback

## Abstract

In our study, we tested a combination of virtual reality (VR) and robotics in the original adjuvant method of post-stroke lower limb walk restoration in acute phase using a simulation with visual and tactile biofeedback based on VR immersion and physical impact to the soles of patients. The duration of adjuvant therapy was 10 daily sessions of 15 min each. The study showed the following significant rehabilitation progress in Control (*N* = 27) vs. Experimental (*N* = 35) groups, respectively: 1.56 ± 0.29 (mean ± SD) and 2.51 ± 0.31 points by Rivermead Mobility Index (*p* = 0.0286); 2.15 ± 0.84 and 6.29 ± 1.20 points by Fugl-Meyer Assessment Lower Extremities scale (*p* = 0.0127); and 6.19 ± 1.36 and 13.49 ± 2.26 points by Berg Balance scale (*p* = 0.0163). *P*-values were obtained by the Mann–Whitney *U* test. The simple and intuitive mechanism of rehabilitation, including through the use of sensory and semantic components, allows the therapy of a patient with diaschisis and afferent and motor aphasia. Safety of use allows one to apply the proposed method of therapy at the earliest stage of a stroke. We consider the main finding of this study that the application of rehabilitation with implicit interaction with VR environment produced by the robotics action has measurable significant influence on the restoration of the affected motor function of the lower limbs compared with standard rehabilitation therapy.

## Introduction

The problem of rehabilitation of acute and subacute cerebrovascular disorders does not lose its relevance at all stages of the disease. The use of modern understanding of neuroplasticity expands rehabilitation opportunities, making them available at different periods of stroke and other neurological disease (Khan et al., 2016; Alia et al., 2017). A comprehensive rehabilitation approach requires rehabilitation from the earliest time of the disease in order to achieve a meaningful recovery of lost functions in the future. From the point of view of motor rehabilitation in early stroke, one of the most important tasks is a patient's verticalization and restoration of overall mobility (Winstein et al., 2016; Powers et al., 2019).

The use of virtual reality (VR) in adjuvant methods of rehabilitation has received much attention recently (Cervera et al., 2018; Maier et al., 2019). Using VR as a tool for repeatedly and naturalistically demonstrating scenes of interaction with real-world objects can influence intercortical interactions in the form of their activation or inhibition in both motor and premotor areas (Léonard and Tremblay, 2006; Laver et al., 2017). There are quite powerful cortico-cortical connections between the occipital, frontal, and parietal lobes, which are used together in the processing of visual, motor, and proprioceptive information (Dum, 2005; Borra and Luppino, 2017). There is evidence that a significant number of neurons in the motor, premotor, and parietal regions are modulated by visual information (Caldara et al., 2004; Ertelt et al., 2007), and virtual avatar movements reveal common parieto-frontal links (Adamovich et al., 2009; Mellet et al., 2009).

Thus, our study is based on previous findings that visual stimulation (in terms of VR) and physical impact to affected limbs contribute to increasing the efficiency of motor rehabilitation of patients with acute cerebrovascular accident.

The purpose of the study was to test the effect of a new method of supplementary motor rehabilitation including VR + robotics therapy on the restoration of the affected walking function in the acute and early recovery periods of ischemic stroke with supratentorial localization.

## Materials and Methods

**Figure 1:**
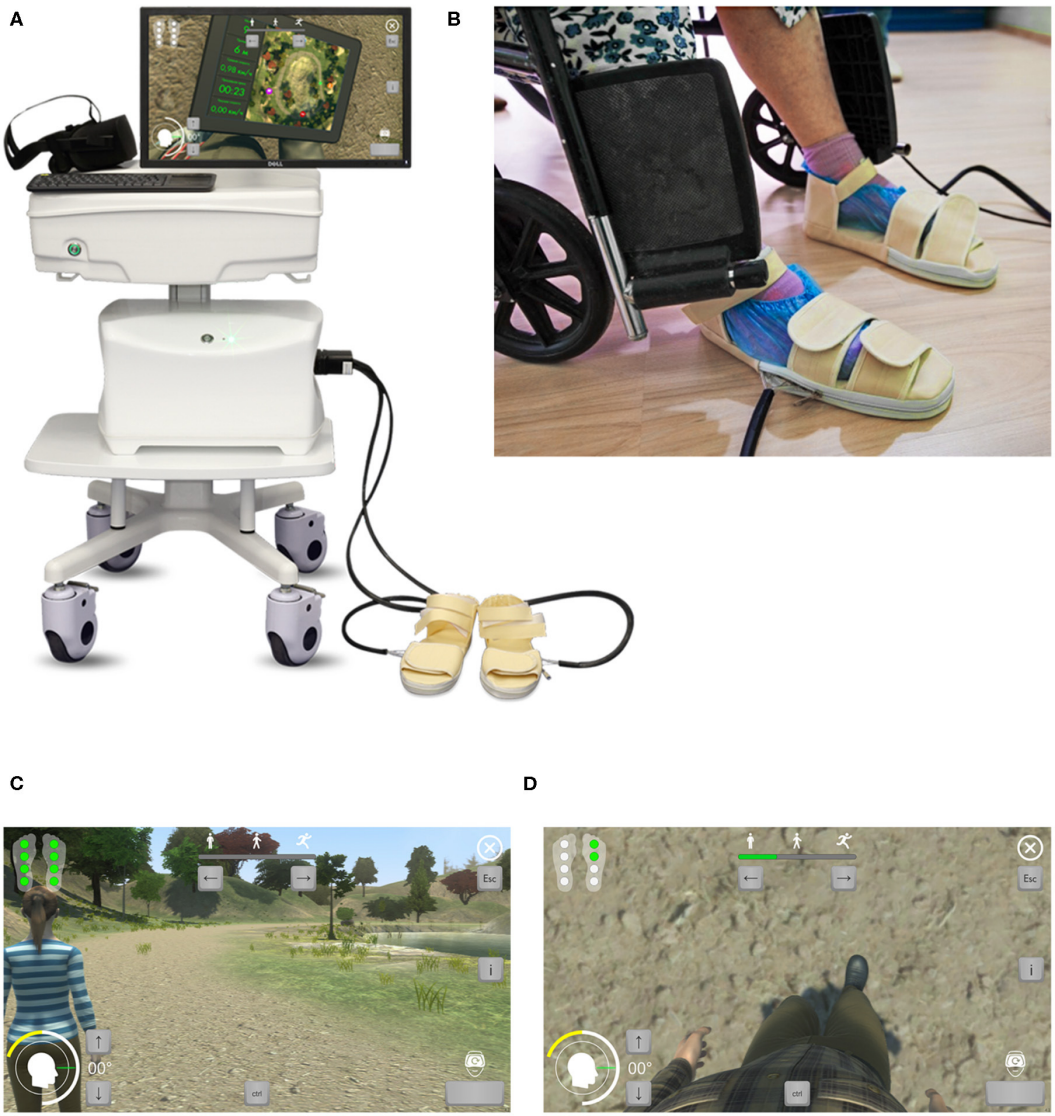
ReviVR rehabilitation walk simulator: **(A)** Equipment overview. **(B)** Patient in a chair with pneumatic orthoses on the feet. **(C)** Patient's avatar and virtual environment overview, third-person view. **(D)** View for a patient in VR-headset, first-person view.

The study continued from November 2018 to October 2019 and included rehabilitation of stroke patients using the ReviVR walk simulator (Figures 1A,B). The ReviVR walk simulator is a proprietary product developed in Samara State Medical University and is protected by Russian and international patents (priority date 29.12.2016; RU2655200C1, WO2018124940A1). The simulator provides immersion in a life-like VR environment and walking imitation with visual and tactile biofeedback based on physical impact—alternate pressing to the soles, synchronized with the “steps” of the avatar in the VR environment.

The patient was wearing a VR headset and two orthoses equipped with four-chamber pneumatic cuffs each fixed on both feet. The chambers in the cuffs were inflated sequentially when a virtual avatar was making a “step.” Such sequential inflation of the four chambers imitated the contact of the sole with the surface during a real walk. The operation of the pressure valves in the chambers (pumping and venting) was synchronized to provide pace of the “walking” to 26 taps per minute on each sole (equivalent to 0.43 “step” per second or one “step” every 2.3 s). The maximal pressure of the chambers on the soles was 0.5 kg/cm^2^ of the plantar surface of the foot. Stimulation of the soles with pneumatic cuffs occurred for both a paralyzed and a healthy limb. The patients saw the virtual environment and their avatar from the “first-person view” in a walking position. By turning a head, the patient could observe the movement of the limbs of the virtual avatar (Figures 1C,D).

Below are the criteria for the inclusion of a patient in the study (in the presence of all of the following):

1. Age 18–80 years with the first-occurred acute ischemic cerebral circulation disorder in the carotid pool.
2. An acute period of cerebrovascular accident: no more than 5 days from the date of stroke.
3. One confirmed focus of ischemic stroke of supratentorial localization according to computerized tomography (CT).
4. Motor disorders in the lower extremities in the form of a central paresis three or less points of a six-point Medical Research Council scale for Muscle Strength (MRC, 1981).
5. Ability and willingness of the patient to comply with the protocol of study requirements.
6. Signed written informed consent.

Below are the exclusion criteria (in the presence of at least one of the following):

1. Explicit cognitive impairment: 10 or less points according to the Montreal Cognitive Assessment scale (MoCA, 1996).
2. Neurological diseases that cause a decrease in muscle strength or an increase in muscle tone in the lower extremities due to any pathology.
3. Clinically significant limitation of the amplitude of passive movements in the lower extremities.
4. Lack of lower limb due to amputation.
5. Any medical condition, including a mental illness or epilepsy, that might affect the interpretation of the results of the study, the conduct of the study, or patient safety.
6. The abuse of alcohol or narcotics within 12 months preceding the moment of inclusion in the study.
7. Treatment with botulinum toxin type A or B in the previous 6 months prior to inclusion in the study.
8. Surgery in the previous 6 months prior to inclusion in the study; for example, abdominal, back, leg, or knee surgery.
9. The severity of the patient's condition according to neurological or somatic status, which does not allow full rehabilitation intervention.
10. Blindness in one or both eyes, or explicit visual impairment more than 20/30 according to Snellen Eye Chart.

The study was performed in the neurology department of Samara Regional Clinical Hospital named after VD Seredavin (53 patients) and in the Research Center of Cerebrovascular Pathology and Stroke, Ministry of Health of the Russian Federation, Moscow (9 patients). The study was approved by the local ethics committee of the Samara Regional Clinical Hospital named after VD Seredavin (protocol #146, 14.03.2018).

All patients included in the study underwent standard rehabilitation. According to their functional state, in addition to medication, they could receive physiotherapy and neuromuscular electrical stimulation (NMES). The choice of methods and the scope of standard therapy by attending clinicians were based on the set of rehabilitation tasks and the functional state of the patient.

Sixty-two patients (M/F, left or right primary hemisphere ischemic stroke) were randomized to the Control (*N* = 27) and Experimental (*N* = 35) groups. Patients' clinical data at the moment of inclusion in the study are presented in Table 1.

**Table 1:** Clinical data of patients included in the study; values presented as mean ± SD.

**Group**	**Gender**	**Stroke pool localization**	**Age**	Points by scale^*^			
	**(M/F)**	**(left/right)**	**(years)**				
				**NIHSS**	**RMI**	**FMA-LE**	**BBS**
Control	14/13	20/7	65.4 ± 1.9	13.0 ± 1.1	2.6 ± 0.4	8.9 ± 1.6	8.9 ± 2.9
Experimental	18/17	22/13	68.1 ± 1.6	12.7 ± 0.7	1.5 ± 0.2	6.4 ± 1.0	2.5 ± 1.1

* Description of scales:

*NIHSS, National Institutes of Health Stroke Scale (0–42 points scale; a lower value is better; used to quantify the impairment caused by a stroke; measured from 0 points—no stroke symptoms to 21–42 points—severe stroke; range 5–15 defined as moderate stroke)*.

*RMI, Rivermead Mobility Index (0–15 points scale; a higher value is better; assesses functional mobility in gait, balance, and transfers after stroke; measured from 0 points—an inability to perform any of the activities to 15 points—full mobility performance)*.

*FMA-LE, Fugl–Meyer Assessment Lower Extremity scale (sections E–F) (0–34 points scale; a higher value is better; used as an index to assess the motor impairment after a stroke; measured from 0 points—hemiplegia to 34 points—normal motor performance)*.

*BBS, Berg Balance Scale (0–56 points scale; a higher value is better; used to determine the ability to safely balance during a series of predetermined tasks; score of <45 indicates that individuals may be at greater risk of falling and score of 56 indicates functional balance)*.

A patient in the Experimental group, initially lying in a bed and then, after 2–3 days, sitting in a chair, received 10 rehabilitation sessions with ReviVR, 15 min each. Thus, the total adjuvant therapy duration was 2.5 h for each patient. Adverse events were monitored throughout the study and were not recorded.

Patients in the Control group were also able to receive rehabilitation with the ReviVR simulator after completion of their participation in the study.

The study completion visit was carried out at the day of discharge of the patient from the hospital, usually on the 21st day. This visit included an assessment on the study scales by an independent neurologist, who was blinded for the patient's rehabilitation group. Rehabilitation performance was evaluated using NIHSS, RMI, FMA-LE, and BBS scales.

Statistical analysis was performed using the STATISTICA data analysis software system, version 12 (StatSoft Inc., 2014, www.statsoft.com).

## Results

It should be noted that patients in both the Control and Experimental groups showed positive rehabilitation dynamics that was observed when assessing the motor function of the lower extremities when performing isolated motor tasks by an affected extremity and during synergistic movements of both lower extremities.

To assess the effectiveness of rehabilitation in the compared groups, we evaluated the progress points (the difference in scores after and before rehabilitation). The progress points were checked for normality of the distribution with the Shapiro–Wilk test. All data showed a non-normal distribution (*p* < 0.02). Assessment of progress in groups was carried out using the two-tailed Mann–Whitney *U* test.

**Figure 2:**
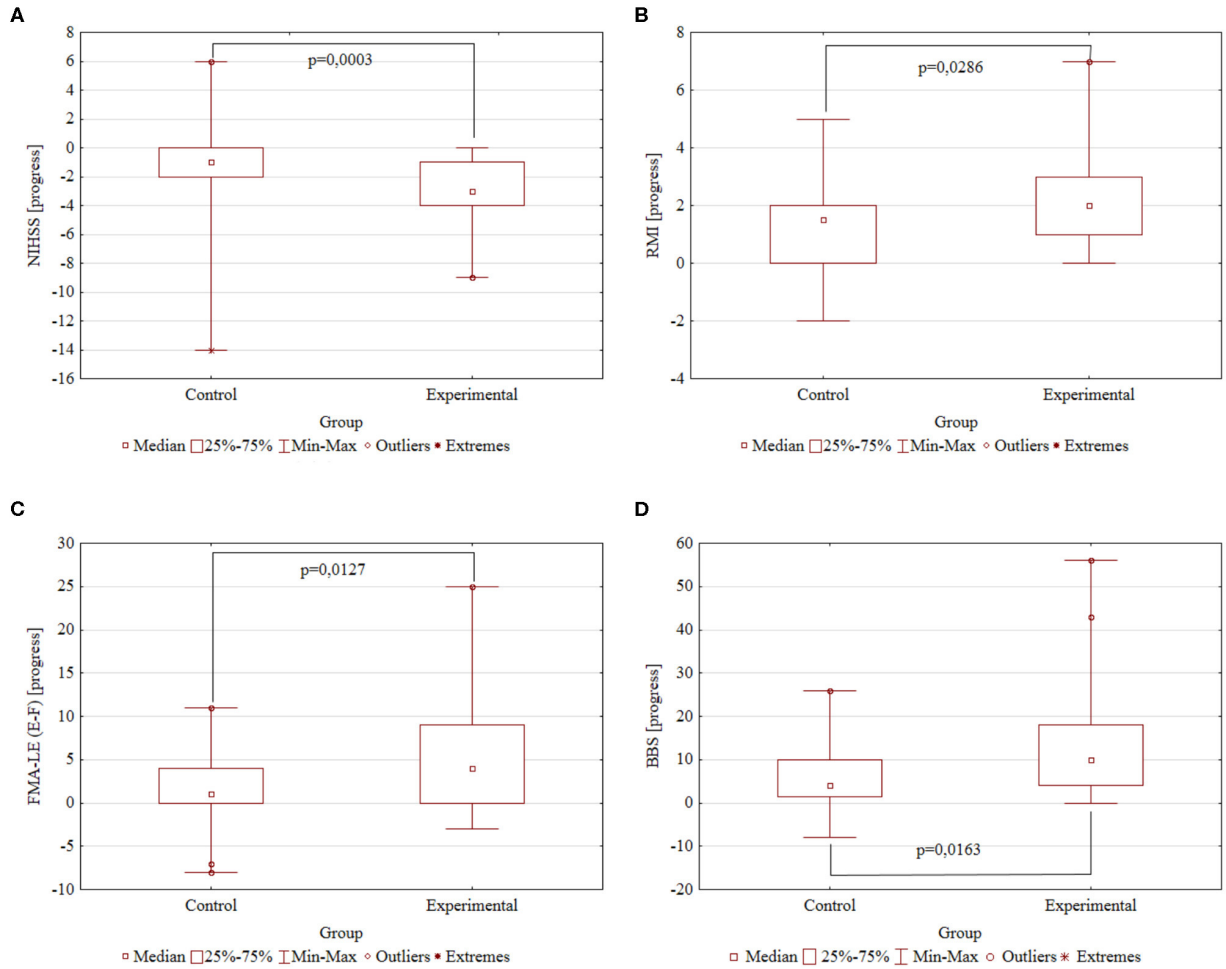
The rehabilitation progresses. Comparison of the Control (*N* = 27) and Experimental (*N* = 35) groups by the study scales. The difference in scores after and before rehabilitation. *P*-value obtained by two-tailed Mann–Whitney *U* test. **(A)** NIHSS progress. **(B)** RMI progress. **(C)** FMA-LE progress. **(D)** BBS progress.

The following are the significant rehabilitation progress points in Control and Experimental groups, respectively: −1.26 ± 0.62 and −2.83 ± 0.32 points by the NIHSS scale (*p* = 0.0003); 1.56 ± 0.29 and 2.51 ± 0.31 points by the Rivermead Mobility Index (*p* = 0.0286); 2.15 ± 0.84 and 6.29 ± 1.20 points by the Fugl–Meyer Assessment Lower Extremities scale (*p* = 0.0127); and 6.19 ± 1.36 and 13.49 ± 2.26 points by the Berg Balance scale (*p* = 0.0163). A higher decrease is better for the NIHSS scale; a higher increase is better for the RMI, FMA-LE, and BBS scales. Summary information on rehabilitation is presented in Table 2 and Figure 2.

**Table 2:** Progress of the rehabilitation; values presented as mean ± SD.

**Group**	**Sample size**	**Progress by scale**			
		**NIHSS**	**RMI**	**FMA-LE**	**BBS**
Control	27	−1.26 ± 0.62	+1.56 ± 0.29	+2.15 ± 0.84	+6.19 ± 1.36
Experimental	35	−2.83 ± 0.32	+2.51 ± 0.31	+6.29 ± 1.20	+13.49 ± 2.26
Statistics *U*	max = 945	235	329	319	305
Effect size Cohen's *d*	–	0.609	0.567	0.681	0.659
*P*-value^*^	–	0.0003	0.0286	0.0127	0.0163

* *Significance of progress scores between the Control and Experimental groups performed by two-tailed Mann–Whitney U test*.

## Discussion, Conclusion, and Further Work

We compared our research with the most similar studies performed in the last 6 years for VR- and robotics-based lower limb rehabilitation (Lee et al., 2014; Xiang et al., 2014; Givon et al., 2015; Ko et al., 2015; Song and Park, 2015; Gibbons et al., 2016; Lo et al., 2017; Darbois et al., 2018; Clark et al., 2019). These studies used the same clinical scales to measure the rehabilitation progress of lower limb function and/or were performed in the acute period of ischemic stroke. Authors also noted an increase in the effectiveness of rehabilitation when using adjuvant therapy.

Evaluating the results of similar studies and our own results, we believe that this influence is due to the impact on motor and premotor areas of neuroplasticity caused by visual, sensory, and cognitive evoked intercortical interactions. The patient's involvement in the virtual environment and the use of the game-like component during the rehabilitation of the lower extremities greatly improve the motor function. Our results are consistent with the findings that patients assimilated the virtual lower limbs as if they were their own legs (Shokur et al., 2016), and we assume that, in our case, there was a similar mechanism of identification (agency) that had a positive effect on neuroplasticity and motor recovery.

The main distinctions of our study are as follows:

- We used the complex activation of neuroplasticity by immersing patients in synchronous visual and sensory passive interaction with virtual environments.
- In our study, we used a relatively simple device to mimic the proprioception sense during the rehabilitation and achieved the progress comparable to the results of interventions with sophisticated robotic equipment that moves the whole lower limbs or the whole body to imitate independent walk.
- The concept of rehabilitation and the design of equipment allow rehabilitation of the following patients:
  − in acute stroke (all 35 patients in the Experimental group);
  − bedridden at the beginning of the study (25 of 35 patients);
  − with severe paresis, diaschisis, or persisting low muscle tone of the lower limb (27 of 35 patients);
  − with afferent and motor aphasia (17 of 35 patients);
  − with restrictions for verticalization due to cardiac arrhythmia, which can cause a cardioembolic stroke and the risk of thromboembolic complications (20 of 35 patients).

Our study shows that an adjuvant post-stroke VR + robotics therapy of the lower extremities in acute phase using interaction via realistic proprioceptive and implicit tactile impacts significantly improves the performance of standard rehabilitation.

We suggest that the use of explicit interaction within walking synergy may show better clinical effects of rehabilitation. We will clarify this hypothesis in our further work.

## Data Availability Statement

All datasets generated for this study are included in the article/Supplementary Material.

## Ethics Statement

The studies involving human participants were reviewed and approved by Local Ethics Committee of the Samara Regional Clinical Hospital named after VD Seredavin (protocol #146, 14.03.2018). The patients/participants provided their written informed consent to participate in this study.

## Author Contributions

AK, AZ, and GI were responsible for the design and the conception of the study. AZ, EK, and YB were responsible for the neurophysiology aspects, organization of patients' enrollment and evaluation, and gathering of the experimental data. AZ and VB contributed to the statistical analysis, manuscript writing, and revision. All authors contributed to the article and approved the submitted version.

## Conflict of Interest

AZ, AK, and EK are employees of Samara State Medical University, which developed the ReviVR simulator used in the study. VB was employed by company IT Universe Ltd. The remaining authors declare that the research was conducted in the absence of any commercial or financial relationships that could be construed as a potential conflict of interest.
